# Glottic insufficiency: the use of fat and fascia grafts

**DOI:** 10.1016/S1808-8694(15)30047-1

**Published:** 2015-10-19

**Authors:** Christiano de Giacomo Carneiro, Domingos Hiroshi Tsuji, Luiz Ubirajara Sennes, João Aragão Ximenes Filho, Rui Imamura

**Affiliations:** a PhD in Otolaryngology - FMUSP, Otolaryngologist.; b Associate Professor of Otolaryngology – FMUSP, Head of the Voice Department HC-FMUSP.; c Associate Professor – Department of Otolaryngology - FMUSP.; d PhD in Otolaryngology - FMUSP, Volunteer physician – Federal University of Ceará.; e PhD in Otolaryngology - FMUSP, Assistant physician – Otolaryngology Department - FMUSP. Otolaryngology Department of the Medical School of the University of São Paulo (FMUSP)

**Keywords:** larynx, graft, autologous, fat, fascia

## Abstract

Glottic insufficiency has been managed since the beginning of the twentieth century. The autologous grafts, as fat and muscular fascia, have shown safety and good results. The authors discuss the advantages and disadvantages of using fat and fascia in the management of glottic insufficiency, regarding historical aspects, inflammatory process and surgical techniques.

## INTRODUCTION

Human voice versatility allows variations in volume, quality and pitch, thus enabling us to express our thoughts and emotions^1^. Even with the growing technological development, we will hardly ever stop communicating. One of the most complex alterations involving the vocal chords is glottic incompetence, or glottic insufficiency. It may be caused by changes in mobility, fibrosis, atrophies or vocal chord “arching”. These may cause, amongst problems such as aspiration and cough, varied degrees of dysphonia^2^. Currently, the search for long lasting solutions or, if possible, permanent to reestablish vocal function with the most quality is one of the goals of phonosurgery.

The attempt to compensate glottic insufficiency started in the XIX century, when Mackenzie proposed arytenoid cartilage stimulation with a galvanic current bearing specific electrodes for cases of unilateral vocal chord paralisis^3^. From the physiopathology stand point, the idea was to catalyze neural recovery, especially for believing that the mobility impairment was a temporary process.

As of the XX century, many surgical procedures were developed in order to rehabilitate glottic valvular and aerodynamic competences. Phonosurgical treatment for paralytic dysphonia starts with the injection of substances in the paraglottic space in order to medialize the paralyzed vocal chord. This was proposed aiming at reorganizing one or more vocal chord properties, such as: tension, length, position, mass and contour.

In 1911, Brunings introduced the injections technique for unilateral vocal chord paralisys^3^. The author himself designed a special syringe for the procedure. He used paraffin, which was abandoned shortly after because of foreign body reactions called “paraffinoma”.

The procedure fell into oblivion until Arnold^4^, in the 60's, resumed the technique of injecting heterologous substances into the vocal chord, such as Teflon. He believed the ideal substance should be well tolerated, “durable” and, should pass through a syringe. Initial results were very promising; however, foreign body reactions, fibrosis, granulomas formation and the possibility of extrusion turned the procedure unfeasible as a definitive treatment. A proof of the effectiveness of this procedure is the very fact that some authors still use it in specific situations. Rosen et al. (1998) proposes the use of Teflon in patients whose life expectance is limited.

Thus, biological implants gained their ground. Bovine collagen is histologically similar to the lamina propria deep layer, and this allows better host integration, besides being easily transported by the injection syringe^5^. Even if no reaction that could seriously impair patients has ever been documented, the use of a bovine protein deep in the lamina propria did not make us feel safe. Previously performed intradermal tests could not guarantee clinical safety^6^.

Human collagen has been recently proposed. The major advantages would be greater durability and no risk of immune response triggering. Initially Ford et al.^7^, and Remacle et al.^8^ later used autologous collagen, in other words, removed from the same body where it would be implanted, while Courey^9^ used homologous collagen taken from cadaver skin. High costs and the lack of FDA (American Food and Drug Administration) approval – hampered its use.

While researchers are still in search of an ideal material, the use of autologous grafts such as fat and muscle fascia is frequent. Initially they showed promising studies, however, their safety and the minimum risk involved have led to their continuous use. In this paper, the authors present a review on the use of fat and muscle fascia to correct glottic insufficiency, and discuss surgical proposals and the histological behavior of each material.

## LITERATURE REVIEW

### Fat

In 1893, Neuber used fat for the first time in an orbit repair procedure after tuberculosis osteitis^3^. In 1992, Mikaelian et al.^10^, were the pioneers in its use in the vocal chord of a patient with unilateral paralysis. Hsiung et al.^11^, in an attempt to better close the glottis and avoid vocal nodule recurrence, proposed fat injection after the surgical removal of nodules. According to Chan and Titze^12^, fat has similar viscosity to that of the lamina propria superficial layer, suggesting that it presents a consistence very close to the ideal one for vocal chord grafting. Fat may be used in glottic insufficiency correction, taken from aspiration and followed by injection through syringes or block transplant. The paraglottic space, lateral to the vocal muscle and the vocal chord vibratory border are the spots the surgeon my use. The choice of technique depends on factors such as glottic incompetence etiology, vocal chord vibration characteristics and the surgeon's experience. Sataloff et al.^13^ studied four patients with vocal chord scars secondary to previous surgical treatment who underwent fat implant. They were the first to describe the building of a pouch in the Reinke space followed by fat injection with a Bruning syringe and large needle to avoid trauma and graft destruction. Despite the subjectiveness of stroboscopic analysis, the authors observed an improvement in vibratory patterns. Duprat et al.^14^ reported their experience with fat use in the treatment of eleven patients with glottic insufficiency caused by groove, open cyst and post-op scar. There was an improvement in vocal and stroboscopic patterns, besides client satisfaction. The technique used was similar to the one used by Sataloff et al.^13^, differing only in the fat transportation form. While they used the syringe, Duprat et al.^14^ used block transportation with conventional microlaryngoscopy instruments. Both did not report the use of sutures or biological glue to improve graft fixation. Vocal chord mobility alterations comprise the major indications for fat injection and should, preferably, be injected through large gauge needles. In order to correct vocal chord fibrosis or scars, it is necessary to use finer needles, and this would not allow fat injection, because its viscosity is high for such procedure. Besides, there would be a destruction of the fat block molecular properties which would hamper graft longevity. The fat injection spot has a direct influence over the final result. According to Imamura et al.^15^, it may be an anecdotal report, but it should be said: fat injected in the thyroarythenoid muscle tends to occupy a cylindrical space along the greater axis of the vocal chord, thus promoting the closure of both the anterior and the posterior glottis. Rosen16 highlights the need for proper exposure, two spot injections (inter-membrane middle third and lateral to the arytenoid cartilage vocal process) and the needle bevel should be turned laterally. The material should be deposited in the paraglottic space.

### Histological Behavior

The use of fat as graft material in laryngology is unpredictable, due to a potential absorption and loss of desired volume. According to the literature, absorption rates are variable, suggesting that fat survival is multifactorial. Among these factors we have harvesting site, and specially, fat tissue graft handling.

We believe there are two different types of subcutaneous fat: a more superficial, ectodermic and closely related to the skin; and another, deeper one, with great genetic influence, difficult to remove with diet alone. The second type is more resistant to lipolysis, and theoretically better for grafting^17^.

Another graft longevity variable is the way by which handling is carried out before injection or block insertion. Excessive fat handling seems to cause greater inflammatory process due to cell rupture and fatty acids release. Aiming at removing fat cells breakdown products, Mikus et al.^18^ proposed fat purification after liposuction and before it is injected. They used dog vocal chords to compare fat injection through two techniques: 1) liposuction; 2) liposuction plus purification. Resorption-related results were better in the long run with liposuction alone. Fibrovascular support loss and fat cell membrane damage are the explanations given by the authors.

Both fat graft size and volume may also influence throughput. Notwithstanding, considering its use in laryngology (Reinke space reconstruction or paraglottic space injection), it is restricted to small spaces, and this factor is not significant.

Another factor that should also be mentioned is the impossibility of having a correct measure of graft throughput. Hörl et al.^19^, compared fat graft throughput when injected on the subcutaneous tissue of 53 patients using MRI, and showed that to correctly estimate it the graft should be far from similar tissues. The authors considered that any graft-related connective tissue or cyst hampers total volume estimates.

Stein et al.^20^ used an animal model to compare local tissue reaction and graft migration after the injection of four different graft types in canine vocal chords, three heterologous (Teflon, silicone and hydroxyapatite) and one autologous (fat). They observed that after six months, hydroxyapatite and fat were the materials that caused the least tissue inflammatory reaction and no migration for cervical lymph nodes. However, the study does not comment upon the throughput of the different types of graft, and it only presents a qualitative analysis of the parameters considered.

Woo et al.^21^ did an experimental study on dog larynxes, using autologous fat implants through larynx microsurgery and slaughtered the animals after 6 weeks. They showed the presence of viable fat cells after this time, besides showing an increase in Reinke's space in comparison with the contralateral vocal chord. Notwithstanding, the short time span between the implant procedure and the animal slaughter could overestimate the beneficial effect of the implanted fat presented by the authors. Still, the lack of a more thorough histology analysis, with a morphometric comparison of graft throughput could prove to be a bias in this study.

### Muscle fascia

The first muscle fascia transplant is believed to have been pioneered by Bruns, who in 1905 corrected a rectal prolapse using fascia lata^22^. It is a graft the otolaryngologist is familiar with, especially those used to patching tympanic membrane holes with it. Thanks to its better adaptation in filling up bi-dimensional spaces, muscle fascia had its use widespread in facial plastic surgery.

Muscle fascias may be laterally injected into the vocal muscle or transplanted in block into a pouch created in the lamina propria. The choice of technique to be used depends on glottic insufficiency etiology. Injection techniques are preferably used in cases of vocal chord unilateral paralysis, while vocal groove and fibrosis are the main indications for fascia implants.

Rihkanen^23^ proposed the injection of “chopped” fascia lata in order to correct glottic insufficiency. Having good initial results, Rihkanen et al.^24^ studied twenty three patients with a diagnosis of unilateral vocal chord paralysis of variable etiology and duration. The fascia was removed under local anesthesia and chopped down into small pieces using a regular scalpel. The injection procedure was carried out under direct microlaryngoscopy, using a pressure syringe, in three or four points. The first one was lateral to the arytenoid vocal process and two or three additional ones were lateral to the thyroarythenoid muscle, up to the vocal chord anterior third. Small amounts of additional fat were used to facilitate syringe transport. Perceptive and acoustic voice analysis, as well as maximum phonation time showed a statistically significant improvement after six months of treatment.

Temporal muscle fascia implant to correct glottic insufficiency caused by vocal groove was described by Tsunoda et al.^25^. The approach used had three basic stages: creating a “pouch” through a careful separation of the adherence between the mucosa and the vocal ligament, fascia preparation and transportation to the vocal chord. In sites where mucosa adherence to the vocal ligament was strong, the “pouch” would penetrate the vocal muscle limits in an attempt to avoid epithelial damage. After a one year average follow up, there was a gradual improvement in maximum phonation time after the third month. Although stroboscopy is a means of subjective analysis, the authors mention improvements in glottic closure and mucosal wave^26^.

### Histological behavior

It is believed that the low metabolic rate and the ultra-structure made up basically of fibrocytes and the collagen extracellular matrix are the main responsible for muscle fascia graft long term survival in the vocal chords. Besides, it properly replaces vibrating tissues^22^ and remains stable within an infected environment such as in chronic ear surgeries^27^.

Muscle fascia histological behavior is still controversial in the literature. In 2000, Rodgers et al.^28^ assessed in an experimental study, the histological behavior of a muscle fascia (lata) injected in dog vocal chords. The animals underwent unilateral section of the recurrent larynx nerve, they were slaughtered after 3, 5, 8, 10 and 12 months and had their larynxes removed and prepared into microscopic slides. The muscle fascia was not identified in any of the cross-sections. On the other hand, Reijonen et al.^27^, also in an experimental study, showed different results. After injecting fascia lata into the paralyzed vocal chords of nine dogs they performed a laryngectomy followed by histological analysis of the slides prepared in 3 and 10 days, 6 and 12 months. The muscle fascia did not cause intense inflammatory reaction nor granuloma formation. Although hystology was quantitative and the final graft throughput was not assessed, it was seen until 12 months post-injection.

### Fascia x Fat

Duke et al.^29^ publish an experimental study histologically comparing fat and muscle fascia. They used eight dogs, which underwent previously purified fat injection, in one of the vocal chords and chopped muscle fascia in the contralateral vocal chord. In the same paper, the authors also carried out a prospective study with forty patients who underwent muscle fascia injection. The major surgical indications were movement alterations, fibrosis or vocal chord arching, and we must stress that six patients had previously undergone fat injection. In the animal study there was no statistically significant difference comparing inflammatory response and final throughput between the grafts (fascia and fat) after 12 weeks. However, in the clinical study, most patients (95%) including those previously subjected to lipo-injection, presented improvements in voice acoustics analysis patterns.

### CONCLUSION

While we still search for the ideal graft, this paper does not propose that either muscle fascia or fat are ideal, however, their safety and low risk of undesirable reactions still maintain their indication in glottic insufficiency.
